# Dietary Fiber-Derived Butyrate Alleviates Piglet Weaning Stress by Modulating the TLR4/MyD88/NF-κB Pathway

**DOI:** 10.3390/nu16111714

**Published:** 2024-05-30

**Authors:** Weikang Huangfu, Jixiang Ma, Yan Zhang, Mengqi Liu, Boshuai Liu, Jiangchao Zhao, Zhichang Wang, Yinghua Shi

**Affiliations:** 1 College of Animal Science and Technology, Henan Agricultural University, Zhengzhou 450046, China; huangfuwk@163.com (W.H.); mjx991010@163.com (J.M.); zhangyan1230228@163.com (Y.Z.); 2019110376@sdau.edu.cn (M.L.); boshuailiu@126.com (B.L.); 2 Henan Key Laboratory of Innovation and Utilization of Grassland Resources, Zhengzhou 450002, China; 3 Henan Forage Engineering Technology Research Center, Zhengzhou 450002, China; 4 Department of Animal Science, Division of Agriculture, University of Arkansas, Fayetteville, AR 72701, USA; jzhao77@uark.edu

**Keywords:** dietary fiber, weaned piglets, gut microbiota, short-chain fatty acid, TLR4/NF-κB signaling pathway, intestinal health

## Abstract

During weaning, piglets are susceptible to intestinal inflammation and impairment in barrier function. Dietary fiber (DF) plays an active role in alleviating weaning stress in piglets. However, the effects of different sources of dietary fiber on the performance of weaned piglets are inconsistent, and the mechanisms through which they affect intestinal health need to be explored. Therefore, in this study, sixty weaned piglets were randomly divided into three treatment groups: basal diet (control, CON), beet pulp (BP), and alfalfa meal (AM) according to the feed formulation for a 28-day trial. The results showed that both AM and BP groups significantly reduced diarrhea rate and serum inflammatory factors (IL-1β and TNF-α) and increased antioxidant markers (T-AOC and SOD), in addition to decreasing serum MDA and ROS concentrations in the AM group. At the same time, piglets in the AM group showed a significant reduction in serum intestinal permeability indices (LPS and DAO) and a substantial increase in serum immunoglobulin levels (IgA, IgG, and IgM) and expression of intestinal barrier-associated genes (*Claudin1*, *Occludin*, *ZO-1*, and *MUC1*), which resulted in an improved growth performance. Interestingly, the effect of DF on intestinal inflammation and barrier function can be attributed to its modulation of gut microbes. Fiber-degrading bacteria enriched in the AM group (*Christensenellaceae_R-7_group*, *Pediococcus* and *Weissella*) inhibited the production of TLR4- through the promotion of SCFAs (especially butyrate). MyD88-NF-κB signaling pathway activation reduces intestinal inflammation and repairs intestinal barrier function. In conclusion, it may provide some theoretical support and rationale for AM to alleviate weaning stress and improve early intestinal dysfunction, which may have implications for human infants.

## 1. Introduction

Weaning is a critical period in the life cycle of mammals when the intestinal function and immune system of young animals are still not fully developed. Sudden changes in diet and social environment can lead to weaning stress, causing intestinal inflammation, damage to the barrier function, diarrhea, growth retardation, and even death. These changes will accelerate the succession of microbial communities [1]. The gut microbiota is the largest and most complex microecosystem in the animal body. It maintains homeostasis under normal conditions and plays a crucial role in promoting the digestion and absorption of nutrients, maintaining normal physiological functions of the gut, regulating immunity, and many other life activities [2]. Dysregulation of the gut microbiome is also one of the causes of intestinal inflammation and diarrhea in newborns and post-weaning animals [3]. Therefore, microbial-based research to minimize the stress faced by young animals at weaning to ensure the stability of their intestinal environment is also a hot issue being explored.

Antibiotics are considered one of the most successful treatments in medicine to reduce the spread of enteric pathogens caused by weaning stress. However, antibiotics have been banned from animal husbandry, where they can spread drug-resistant pathogens, affect the microbial balance of organisms, and cause problems with food residues [4]. Therefore, finding alternatives to antibiotics to reduce intestinal inflammation and barrier function damage induced by weaning stress is crucial for animal feeding and food safety.

Nutritional modification is used as one of the effective tactics to improve the health and growth of weaned piglets [5], and the use of specific dietary ingredients, such as dietary fiber (DF), has been widely investigated as a way to prevent and treat diseases by modulating the gut microbiota [6]. According to its physical and chemical properties, DF can be divided into soluble dietary fiber (SDF) and insoluble dietary fiber (IDF) [7], in which beet pulp (BP), composed of SDF, such as pectin and dextran [8], has been widely used in studies on weaned piglets [8,9,10]. Alfalfa meal (AM) is a slowly fermentable IDF composed of cellulose and lignin [11]. Studies have shown that AM plays a vital role in improving the gut health of piglets, but the exact mechanism still needs to be explained [12,13]. However, different sources of DF play different roles in improving intestinal microbial structure and metabolite composition [7]. Short-chain fatty acids (SCFAs), as one of the essential products of DF fermentation, play a crucial role in reducing intestinal inflammation by enhancing the intestinal ecosystem, providing energy to the organism, and modulating the inflammatory signaling cascade by inducing immunity [14]. As potentially bioactive molecules, SCFAs mainly initiate immune responses and regulatory mechanisms to repair the intestinal mucosa and attenuate inflammatory damage, mainly through the underlying signaling cascade mediated by the G protein-coupled receptor (GPR)/Toll-like receptor (TLR), which has become an emerging theme of modern interest [15]. Thus, the appropriate intake of DF in the diet and the mechanism mediated by the microbial-SCFA level are essential for regulating the intestinal health of the host.

Meanwhile, as early-weaned piglets are prone to microecological dysregulation and diarrhea, pigs are highly similar to humans in anatomy, physiology, nutrient metabolism, and microbial ecosystems [16]. Hence, using piglet weaning stress as a model of inflammation, this study first investigated the effects of BP and AM on the growth performance of weaned piglets. It also explored the key microbial-level-based mediating roles of DF in intestinal inflammation and intestinal barrier regulation and their mechanisms. In addition, this study may provide some theoretical support and a basis for DF nutrition to improve intestinal dysfunction in infants and young children.

## 2. Materials and Methods

The animal experimental procedures involved in this study were conducted following the standards of the Animal Welfare and Ethics Committee of Henan Agricultural University (Zhengzhou, China) (approval number: HENAU-2024-021).

### 2.1. Chemical Analysis

Dry matter (DM) and crude protein (CP) of BP and AM fiber products were analyzed according to AOAC, 2007. Neutral detergent fiber (NDF), acid detergent fiber (ADF), SDF, and IDF were determined by a fiber analyzer (Ankom Technology, Macedon, NY, USA) according to the method described by Van Soest et al. [17]. The total dietary fiber (TDF) content was calculated as the sum of SDF and IDF. The results are shown in Table S1.

### 2.2. Animals and Treatment

Sixty similarly sized 28-day weaned piglets (Duroc × Landrace × Large, BW = 8.75 ± 0.33 kg) were selected and randomly divided into three groups of four replicates (pens) of five piglets each, as follows: (1) corn–soybean meal basal diet without antibiotics (control group, CON); (2) 5% BP replacement of basal diet (BP); and (3) 5% AM replacement of basal diet (AM). All diets were formulated to meet the nutritional needs of weaned piglets according to the recommendations of the NRC (2012). The diet composition and nutrient levels of all piglets during the trial period are shown in Table 1. The trial lasted 35 days, including a pre-test period of 7 days and a test period of 28 days, during which all piglets were free to drink and feed.

### 2.3. Growth Performance and Diarrhea Rate

During the trial, the daily feed intake of each replicate was accurately recorded, the health condition was observed, and the weight of each piglet was recorded at the beginning and end of the trial on an empty stomach. The average daily feed intake (ADFI), average daily gain (ADG), and feed-to-gain ratio (F:G) for the whole trial cycle were calculated.

The number of piglets with diarrhea in each group was calculated according to the formula of Wu et al. [18] for each replicate (pen); diarrhea rate (%) = cumulative number of piglets with diarrhea/number of days of counting/number of piglets in each replicate (5) × 100%.

### 2.4. Sample Collection

At the end of the experiment, one piglet was randomly selected from each replicate, and 10 mL of blood was collected from the anterior elbow vein, centrifuged (3500 rpm, 15 min) to obtain serum, and stored at −20 °C. Subsequently, the piglets were anesthetized intravenously with sodium pentobarbital (80 mg/kg body weight). After euthanasia, the abdominal cavity was opened with a scalpel to expose the viscera, and the intestinal segments were identified and ligated. Individual organs were weighed separately; jejunum and ileum were collected and stored in 4% paraformaldehyde solution for intestinal histomorphometry; intestinal tissues (ileum and colon) were collected for subsequent analyses; and intestinal contents (ileum and colon) were collected for microbiota composition and SCFAs analysis. 

### 2.5. Measurement of Organ Indexes

Liver and spleen organs were collected, and organ index was assessed according to the formula: organ index (%) = organ weight/body weight × 100%.

### 2.6. Serum Parameters Analysis

Barrier factors: Lipopolysaccharide (LPS), diamine oxidase (DAO) activity, D-lactic acid (D-LA) level, and cortisol content in serum were determined by enzyme-linked immunosorbent assay (ELISA) kits (Shanghai Enzyme Linked Biotechnology Co., Ltd., Shanghai, China) following the manufacturer’s instructions.

Immune responses: serum concentrations of inflammatory cytokines, including interleukin 1β (IL-1β), interleukin 10 (IL-10), and tumor necrosis factor-alpha (TNF-α), as well as immunoglobulins, including IgA, IgG, and IgM, were detected using a pig-specific ELISA kit (Shanghai Enzyme Linked Biotechnology Co., Ltd., Shanghai, China).

Antioxidant capacity: antioxidant indices, including total antioxidant capacity (T-AOC), total superoxide dismutase (T-SOD), catalase (CAT), glutathione peroxidase (GSH-Px), and malondialdehyde (MDA), were determined in serum using a porcine-specific kit (Nanjing Jiancheng Bioengineering Institute, Nanjing, China).

### 2.7. Intestinal Morphology Analysis

Intestinal developmental morphology was observed and scored by hematoxylin and eosin (H&E) staining. Tissue samples were fixed in 4% paraformaldehyde for 24 h, dehydrated with 50% to 100% ethanol, and embedded in paraffin. Intestinal samples were cut into 3 μm sections using a microtome and stained with hematoxylin and eosin. After sealing through cedar oil, the tissue was photographed under a microscope. At least 10 intact and correctly oriented units of villi and crypts were observed in each section; villus height and depth were measured, and the villus–crypt ratio was calculated.

### 2.8. Real-Time Quantitative Polymerase Chain Reaction (RT-qPCR)

RNA was isolated from ileal and colonic tissues using Trizol reagent (Cwbio, Taizhou, China) and quantified by NanoDrop 2000c spectrophotometer (Thermo Fisher Scientific, Waltham, MA, USA). Total RNA was reverse transcribed into complementary DNA (cDNA) using HiScript Ⅲ RT SuperMixR323 (Vazyme, Nanjing, China). RT-qPCR was performed on a 7900 HT real-time PCR system using ChamQ Universal SYBR qPCR Master Mix (Vazyme, Nanjing, China). According to the kit instructions, the RT-qPCR process was performed on a Roche Lightcycler96 system. The data were analyzed by the comparative Ct method, and the transcript levels of the target genes were normalized to the mRNA levels of the internal reference gene GAPDH. The primer sequences were synthesized by Sunya Biotechnology Co., Ltd., Hangzhou, China (Table S2).

### 2.9. Western Blotting

Western blotting procedures were conducted by our previously described methods [19]. Colon tissues were lysed using radio immunoprecipitation assay (RIPA) lysis buffer containing protease inhibitor (Solarbio, Beijing, China) and phosphatase inhibitor (Solarbio, Beijing, China). Equal amounts of proteins were separated on a 10% sodium dodecyl sulfate–polyacrylamide gel electrophoresis (SDS-PAGE) gel and transferred to a polyvinylidene fluoride membranes (PVDF) membrane (03010040001, Roche, Basel, Switzerland). The membranes were blocked with NcmBlot blocking buffer (NCM Biotech, Suzhou, China) for 10 min at room temperature, and then the primary antibodies were incubated overnight at 4 °C. The following antibodies were used in this study: Toll-like receptor 4, TLR4 (A5258, ABclonal, Woburn, MA, USA), myeloid differentiation factor 88, MyD88 (23230-1-AP, Proteintech, Wuhan, China), nuclear factor kappa B p65, NF-κB p65 (10745-1-AP, Proteintech, Wuhan, China), Phospho-NF-κB p65, P-NF-κB p65 (82335-1-RR, Proteintech, Wuhan, China), and β-actin (A5441, Sigma-Aldrich, St. Louis, MO, USA). Then, horseradish peroxidase-conjugated IgG was added and incubated at 37 °C for 2 h. A chemiluminescence kit (P0018FM, Beyotime, Shanghai, China) was added, and protein bands were visualized with a chemiluminescence imager (C300, Azure Biosystems, Dublin, CA, USA). The results were quantified using Image J v1.8.0 software (NIH, Bethesda, MD, USA).

### 2.10. 16S rRNA Gene Sequencing and Analysis

The microbiota in piglet intestinal contents was analyzed by 16S rRNA sequencing. Briefly, total genomic DNA was extracted from contents samples using the Fecal DNA kit (Omega Bio-tek, Norcross, GA, USA). Subsequently, the bacterial 16S rRNA gene V3-V4 hypervariable regions were amplified using primers 338F (5′-ACTCCTACGGGAGGCAGCAG-3′) and 806R (5′-GGACTACHVGGGTWTCTAAT-3′), with the following program: 3 min denaturation at 95 °C; 27 cycles of 30 s at 95 °C, 30 s annealing at 55 °C, and 45 s elongation at 72 °C; and a final extension at 72 °C for 10 min. PCR reactions were performed in triplicate, with each 20 μL reaction mixture containing 4 μL of 5× FastPfu Buffer, 2 μL of 2.5 mM dNTPs, 0.8 μL of each primer (5 μM), 0.4 μL FastPfu Polymerase, and 10 ng template DNA. Amplicon libraries were sequenced, and the number and size of the amplicon libraries were assessed using the AxyPrepDNA Gel Recovery Kit (AxygenBiosciences, Union City, CA, USA). When sample purity met the standard requirements, sequencing was performed on the Illumina MiSeq platform (San Diego, CA, USA) following standard operating procedures by Majorbio Bio-Pharm Technology Co., Ltd. (Shanghai, China). Sequences with 97% similarity were classified into operational taxonomic units (OTUs) using Uparse software (http://www.drive5.com/uparse/, accessed on 1 May 2022, version 11). Species classification was annotated for each sequence using the RDP classifier (https://sourceforge.net/projects/rdp-classifier/, accessed on 1 May 2023, v2.13), and species comparisons were performed through the Silva 138 database (https://www.arb-silva.de/, accessed on 1 May 2023) to obtain species classification information, and sequencing data were analyzed by genomics v1.8.1 software (Visual Genomics Soft). To ensure data quality, samples with reads below 10 were filtered out by removing low-abundance OTUs.

For the analysis of α-diversity (Shannon and Simpson) and β-diversity (principal coordinates analysis, PCoA, and non-metric multidimensional scaling, NMDS), LEfSe using linear discriminant analysis (LAD > 2.5) was performed through the Majorbio cloud platform (www.majorbio.com, accessed on 1 November 2023), differential microbes were identified using the Kruskal–Wallis H-test, and Spearman’s correlation test tested the relationship between the core microbes and the phenotypic data. The network diagrams were plotted by Cytoscape. The original reads were deposited with the National Center for Biotechnology Information under project number PRJNA1034756.

### 2.11. SCFAs’ Measurement

According to a previous description, the concentration of SCFAs in piglet colonic contents was determined by ion chromatography [20]. Briefly, approximately 0.5 g of colonic contents was added to 10 mL of ultrapure water, processed in an ultrasonic water bath for 30 min, and then centrifuged at 12,000 rpm for 10 min at 4 °C. Subsequently, 1 mL of the supernatant was taken, 0.2 mL of 25% metaphosphoric acid was added, and the supernatant was allowed to stand at 4 °C for 30 min before being centrifuged at 10,000 rpm for 10 min. Finally, 100 µL of the supernatant was diluted 10-fold with deionized water and filtered through a 0.22 µm membrane. The filtered fluids were measured by an ICS-5000 ion chromatography system (Dionex, Sunnyvale, CA, USA) with an Aminex AH11-HC column (Biorad, Hercules, CA, USA). The SCFAs’ content of the samples was calculated from the standard curve and peak area.

### 2.12. Statistical Analysis

The results are expressed as mean ± Standard Error of the Mean (SEM), * *p* < 0.05; ** *p* < 0.01; *** *p* < 0.001; *p* < 0.05 means a trend to show significant difference. Differences between groups were analyzed by one-way analysis of variance using GraphPad Prism v8.0.1 software (San Diego, CA, USA), followed by Tukey’s multiple comparisons tests.

## 3. Results

### 3.1. Effect of Different Sources of DF on Growth Performance and Weaning Stress

**Figure 1:**
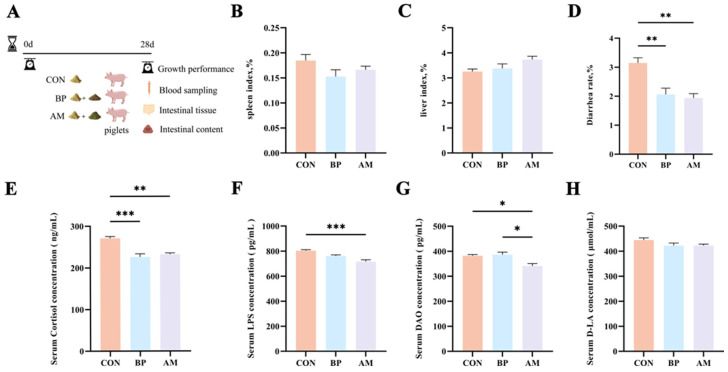
Effects of different sources of DF on weaning stress and diarrhea rate. (**A**) Design of animal rearing experiment and sample collection. (**B**) The spleen index or (**C**) liver index of piglets by tissue weight/body weight (g/kg). (**D**) The diarrhea rate of piglets. (**E**) The cortisol (**F**) LPS (**G**) DAO or (**H**) D-LA levels of serum in piglets. Data are expressed as the mean ± SEM. * *p* < 0.05; ** *p* < 0.01; *** *p* < 0.001. n = 4.

A 28-day feeding experiment was conducted to investigate the effects of BP and AM on weaning stress and piglet growth performance (Figure 1A). There was no significant change in ADG and F:G and a significant decrease in ADFI in the BP group compared to the CON group (*p* > 0.05) (Table 2). On the contrary, compared with the CON group, the AM group showed an increasing trend in final body weight (*p* = 0.08), a significant increase in ADG, and a significant decrease in F:G (*p* < 0.05) (Table 2). In addition, there were no differences in spleen and liver indexes among the groups (*p* > 0.05) (Figure 1B,C).

Subsequently, we measured the effects of weaning stress and intestinal permeability. The diarrhea rate and cortisol concentration in the BP and AM groups were significantly lower than in the CON group (*p* < 0.05) (Figure 1D,E). In addition, LPS and DAO levels were significantly lower in the AM group compared to the CON group (*p* < 0.05) (Figure 1F,G). There was no difference in D-LA between the groups (*p* > 0.05) (Figure 1H).

### 3.2. Different Sources of DF Improve Serum Immunity and Antioxidant Capacity

**Figure 2:**
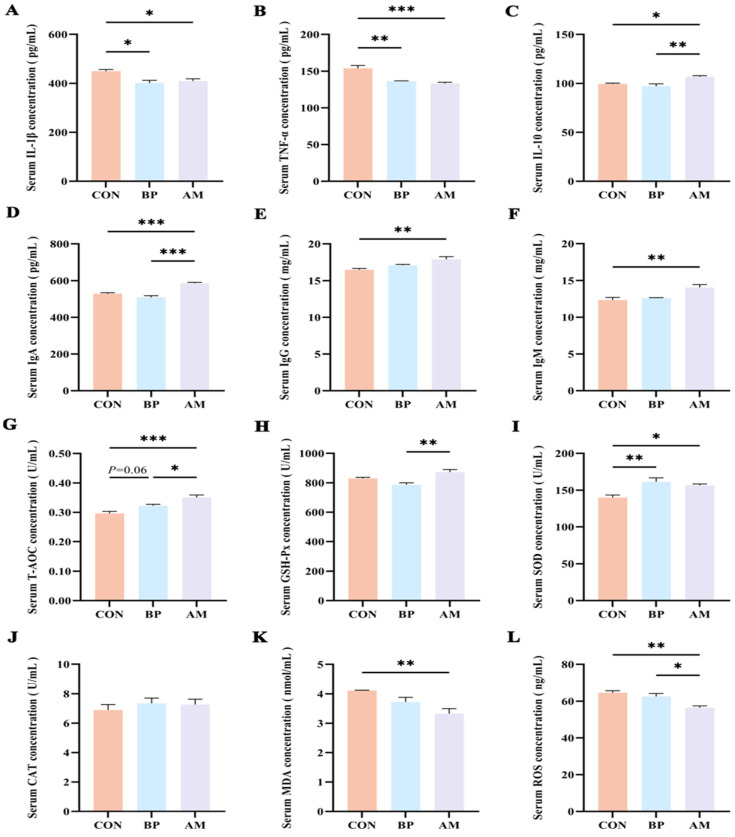
Different sources of DF alleviate serum inflammatory response and oxidative stress. (**A**–**C**) Serum inflammatory factors (IL-1β, TNF-α, IL-10). (**D**–**F**) Immune globulin contents (IgA, IgG, IgM). (**G**–**L**) Serum antioxidant indexes (T-AOC, GSH-Px, SOD, CAT, MDA, ROS). Data are expressed as the mean ± SEM. * *p* < 0.05; ** *p* < 0.01; *** *p* < 0.001. n = 4.

In order to detect the effects of BP and AM on the immune response of weaned piglets, we examined serum inflammatory factor levels and immunoglobulins content (Figure 2). Compared to the CON group, the BP and AM groups significantly decreased the concentration of IL-1β and TNF-α in serum (*p* < 0.05) (Figure 2A,B). In addition, the concentration of the anti-inflammatory cytokine IL-10 and immunoglobulins (IgA, IgG, and IgM) was significantly increased in the AM group compared with the CON group (*p* < 0.05) (Figure 2C–F).

Subsequently, we measured the antioxidant capacity indices in serum. The T-AOC concentration in the AM group was significantly higher than in the CON group (*p* < 0.05). Similarly, the T-AOC concentration in the BP group showed an increasing trend (*p* = 0.06) (Figure 2G). In addition, SOD concentration was significantly higher in the BP and AM groups compared with the CON group (*p* < 0.05) (Figure 2I). Also, MDA and ROS concentration was significantly lower in the AM group (*p* < 0.05) (Figure 2K,L), while other antioxidant indices (GSH-Px, CAT) were not different compared with the CON group (*p* > 0.05) (Figure 2H,J).

### 3.3. Effects of Different Sources of DF on Intestinal Development and Barrier Function

**Figure 3:**
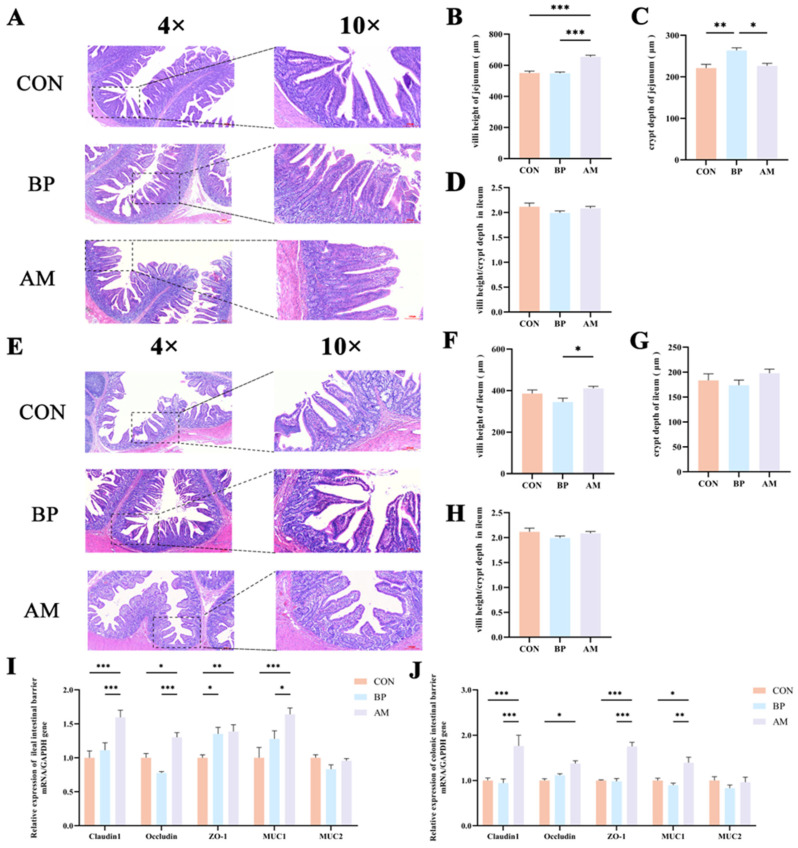
Effects of different sources of DF on intestinal development and barrier function. (**A**) The morphology images of jejunum. (**B**) Villi height, (**C**) crypt depth, (**D**) villi height/crypt depth value of jejunum. (**E**) The morphology images of the ileum. (**F**) Villi height, (**G**) crypt depth, (**H**) villi height/crypt depth value of ileum. (**I**) The relative gene expression levels of barrier function in ileal tissues. (**J**) The relative gene expression levels of barrier function in colon tissues. The × represents a magnification of the objective lens. Data are expressed as the mean ± SEM. * *p* < 0.05; ** *p* < 0.01; *** *p* < 0.001. n = 4.

In order to examine the effects of BP and AM on intestinal development, we observed intestinal morphology and measured the villus height and crypt depth (Figure 3). In the jejunum, villus height and villus height/crypt depth were significantly higher in the AM group than in the CON and BP groups (*p* < 0.05) (Figure 3B). However, the BP group had significantly increased crypt depth and significantly decreased villus height/crypt depth compared with the CON group (*p* < 0.05) (Figure 3C,D). In addition, in the ileum, the villus height was significantly lower in the BP group than in the AM group (*p* < 0.05) (Figure 3F). There were also no significant differences between the other indices (*p* > 0.05) (Figure 3G,H).

Intestinal barrier function and host defense peptides (HDPs) were further detected to reflect piglet defense ability and health status. By measuring the barrier protein-related genes in the ileum and colon, the results showed that the relative expression levels of *Claudin1*, *Occludin*, *ZO-1*, and *MUC1* genes in the AM group were significantly higher than those in the CON group (*p* < 0.05) (Figure 3I,J). Similarly, the expression of the *NKlysin* gene in the AM group was also significantly higher than in the CON group (*p* < 0.05) (Figure S1).

### 3.4. Effects of Different Sources of DF on Ileal Microbiota of Weaned Piglets

**Figure 4:**
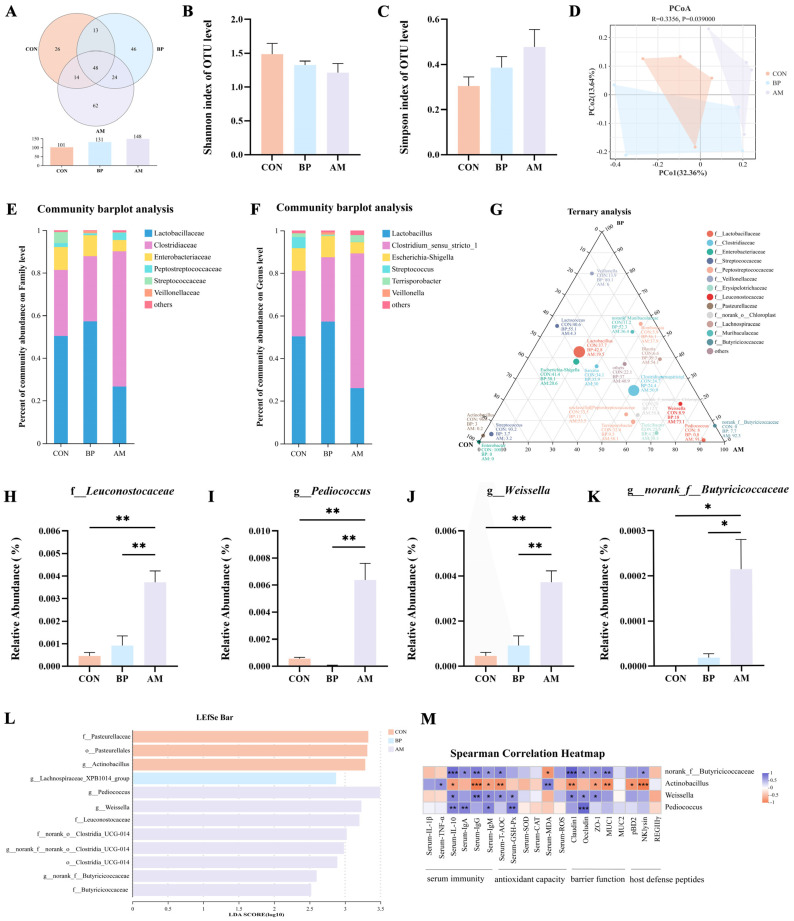
Effects of different sources of DF on ileal microbiota. (**A**) Venn diagram of OTU levels. (**B**,**C**) The α diversity of microbiota (Shannon, Simpson). (**D**) The β diversity of microbiota. (**E**,**F**) Microbial community composition (family and genera level, the abundance of communities showing abundances greater than 0.01). (**G**) Ternary diagram showing the composition and distribution of the top 20 dominant species in abundance. (**H**) Differential microbiota in the top 10 abundances at the family level. (**I**–**K**) Differential microbiota in the top 20 abundances at the genus level (*Pediococcus*, *Weissella*, and *norank_f__Butyricicoccaceae*). (**L**) LDA demonstrates biomarkers in the microbiota among treatments (LDA > 2.5, *p* < 0.05). (**M**) Heat map of core microbiota and intestinal health-related indicators. Data are expressed as the mean ± SEM. * *p* < 0.05; ** *p* < 0.01; *** *p* < 0.001. n = 4.

The effects of BP and AM on gut microbial community structure were analyzed using 16S rRNA gene sequencing. A total of 547,853 high-quality reads (average 45,654) were obtained from 12 ileum content samples. Subsequently, the dilution curve showed that as the sequencing amount increased, the species’ richness gradually tended to be flat, indicating enough sequencing depth to study the dominant microbial (Figure S2). The Venn diagram showed that the number of OTUs in the ileum samples of the AM group was the largest (Figure 4A). The α-diversity indices showed no significant changes in the abundance and diversity of the microbial communities in the three groups (*p* > 0.05) (Figure 4B,C). β-diversity analyses based on unweighted unifrac distances from PCoA (Figure 4D) and NMDS (Figure S3A) showed significant differences between the three groups of communities (*p* < 0.05). Through separate analysis by PCoA, it was revealed that the AM group had the more extraordinary ability to modulate the gut microbiota (*p* < 0.05) (Figure S3B). The abundance of communities at the family and genus level is shown in Figure 4E,F, suggesting that DF affects the composition of microbes. The ternary diagram showed that *Actinobacillus*, *Enterobacter*, and *Streptococcus* were mainly enriched in the CON group. *Veillonella* was mainly present in the BP group, and microbiota such as *norank_f__Butyricoccaceae*, *Pediococcus*, and *Weissella* were more abundant in the AM group (*p* < 0.05) (Figure 4G). Detailed analyses of communities with significant changes in relative abundance were performed. The AM group *Leuconostocaceae* was significantly higher at the family level than in the CON group (*p* < 0.05) (Figure 4H). At the genus level, the AM group significantly increased the relative abundance of *Pediococcus*, *Weissella*, and *norank_f__Butyricicoccaceae* compared to the CON group (*p* < 0.05) (Figure 4I–K). The biomarkers in the microbiota of different treatment groups were analyzed by linear discriminant analysis (Figure 4L). A total of six genus-level microbial groups were identified, among which the AM group was enriched with the most significant number of microbes, as *Pediococcus*, *Weissella*, and *norank__f__Butyricoccaceae* (*p* < 0.05). Subsequently, the phylogenetic tree revealed that *Actinobacillus* was more closely related to *Escherichia-Shigella* and *Pasteurella*; *Pediococcus* was more closely related to *Lactobacillus* and *Weissella* (Figure S3C). A heat map of the correlation network revealed that *Actinobacillus* was significantly negatively correlated with *Weissella*, *norank__f__Butyricicoccaceae* (*p* < 0.05) (Figure S3D). We developed heat maps based on Spearman’s correlation analysis, and showed similar correlations for *Pediococcus*, *Weissella*, and *norank_f__Butyricoccaceae*, which showed significant positive correlations with serum immunityindicators (IL-10, IgM) and the gut barrier indicator *Occludin* (*p* < 0.05). In contrast, the results for *Actinobacillus* were the opposite (*p* < 0.05) (Figure 4M).

### 3.5. Effects of Different Sources of DF on Colonic Microbiota of Weaned Piglets

**Figure 5:**
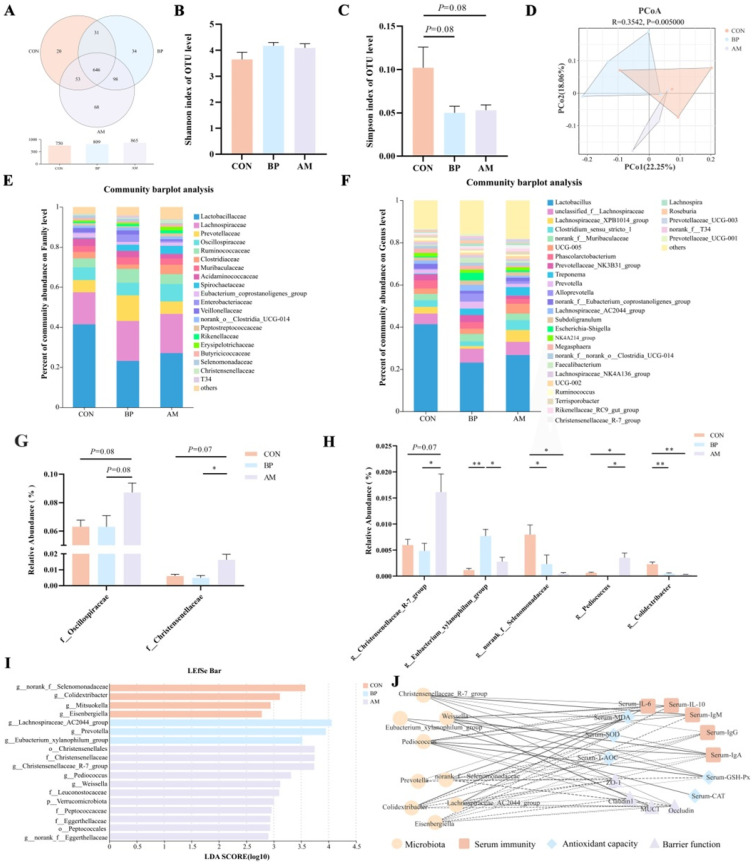
Effects of different sources of DF on colon microbiota. (**A**) Venn diagram of OTU levels. (**B**,**C**) The α diversity of microbiota (Shannon, Simpson). (**D**) The β diversity of microbiota. (**E**,**F**) Microbial community composition (family and genera level, the abundance of communities showing abundances greater than 0.01). (**G**) Differential microbiota in the top 20 abundances at the family level. (**H**) The top 5 microbes in terms of genus-level differences. (**I**) LDA demonstrates biomarkers in the microbiota among treatments (LDA > 2.5, *p* < 0.05). (**J**) Network analysis of enriched microbiota and intestinal health-related indicators: solid lines indicate positive correlations, and dotted lines indicate negative correlations. Data are expressed as the mean ± SEM. * *p* < 0.05; ** *p* < 0.01. n = 4.

**Figure 6:**
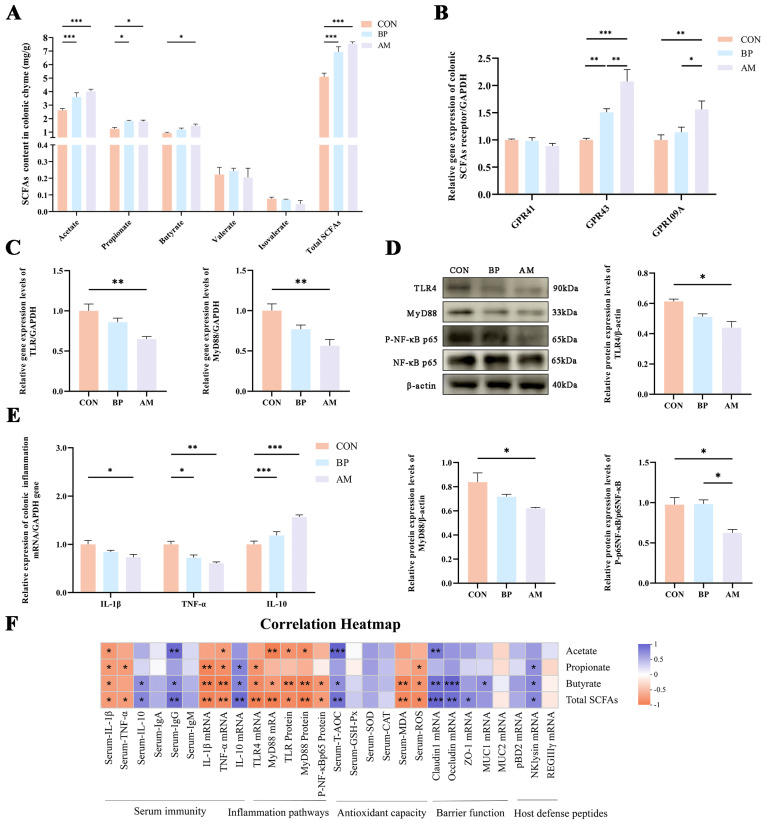
Dietary fiber-derived SCFAs improve Gut health by modulating relevant receptors and TLR/MyD88/NF-κB pathways. (**A**) Effect of fiber source on SCFAs. (**B**) The relative gene expression levels of SCFAs receptors. (**C**) Gene expression of inflammation-related pathways. (**D**) Protein expression of inflammation-related pathways. (**E**) The relative gene expression levels of inflammatory factors in colon tissues. (**F**) Heat map analysis of correlations between SCFAs and HDPs, inflammatory factors and their pathways, and the intestinal barrier. Statistical significance determined by one-way ANOVA with post hoc Tukey’s multiple comparisons tests. Data are expressed as the mean ± SEM. * *p* < 0.05; ** *p* < 0.01; *** *p* < 0.001. n = 4.

We further investigated the effect of different DF on the colonic microbiota. A total of 684,496 high-quality reads (average 57,041) were obtained from 12 colon content samples. Similarly, the colon dilution curve indicates sufficient sequencing depth to study the dominant microbiota (Figure S4). The Venn diagram showed the composition of the three groups of OTUs, indicating that the AM group had the most abundant OTUs (Figure 5A). There was no difference in the Shannon index among the three groups by α diversity (*p* > 0.05). However, there was a decreasing trend in the Simpson index in the BP and AM groups compared to CON (*p* = 0.08) (Figure 5B,C), indicating a tendency of elevated microbial diversity in the BP and AM groups. According to PCoA (Figure 5D) and NMDS (Figure S5A), there was a significant difference among the three groups of communities (*p* < 0.05). Subsequent analysis based on PCoA alone revealed that (Figure S5B), consistent with the ileal results, the addition of AM significantly affected the gut microbiota’s composition. The abundance of communities at the family and genus level is shown in Figure 6E,F, suggesting that DF affects the composition of microbes. The microbes with significant changes in relative abundance were analyzed. At the family level, the relative abundance of *Oscillospiraceae* in the AM group tended to increase compared with that of the CON and BP groups (*p* = 0.08). The *Christensenellaceae* in the AM group also tended to increase compared with the CON group (*p* = 0.07) and was significantly higher than in the BP group (*p* < 0.05) (Figure 5G). At the genus level, the AM group *Christensenellaceae_R-7_group* was significantly higher than in the BP group and tended to be elevated compared to the CON group (*p* = 0.07). *Eubacterium_xylanophilum_group* in the BP group had a relative abundance significantly higher than the CON and AM groups (*p* < 0.05). In contrast, the BP and AM groups significantly reduced the relative abundance of *norank_f__Selenomonadaceae* and *Colidextribacter* compared with the CON group (*p* < 0.05). Also, the *Pediococus* of the AM group was significantly higher than that of the CON and BP groups (*p* < 0.05) (Figure 5H). The LDA analysis of the three groups identified a total of 11 marker microbes at the genus level, including *norank_f__Selenomonadaceae*, *Colidextribacter*, *Eubacterium_xylanophilum_group*, *Christensenellaceae_R-7_group*, *Pediococcus*, and *Weissella* (Figure 5I). In addition, phylogenetic evolutionary trees indicate closer affinities between microbes at the same family level, e.g., *norank_f__Selenomonadaceae*, *Selenomonas*, and *Anaerovibrio* belong to the *Selenomonadaceae* family (Figure S5C). A heat map of the correlation network among microbes was constructed, and it was found that there was also a significant positive correlation between the AM groups *Christensenellaceae_R-7_group*, *Pediococcus*, and *Weissella*, and the CON groups *norank_f__Selenomonadaceae* and *Colidextribacter* (Figure S5D). Finally, correlation network diagrams were constructed based on Spearman’s analysis, and similar correlations were found for *Christensenellaceae_R-7_group*, *Pediococcus*, and *Weissella*, which were manifested by the correlation with serum immunity indicators (IL-10, IgA, IgG, and IgM), antioxidant indices GSH-Px, and intestinal barrier-related indices (*ZO-1*, *Occludin*, and *MUC1*) (*p* < 0.05). In contrast, the results for *norank_f__Selenomonadaceae* and *Colidextribacter* were the opposite (*p* < 0.05). In addition, *Eubacterium_xylanophilum_group* was significantly positively correlated with antioxidant indices, CAT and SOD (*p* < 0.05) (Figure 5J).

### 3.6. DF Alleviates Weaning Stress by Regulating the TLR/MyD88/NF-κB Pathway through Gut-Microbe-Derived SCFAs

DF fermentation produces SCFAs, which participate in regulating intestinal homeostasis. Therefore, we determined the concentration of SCFAs in the colonic contents and the relative expression of their receptors in tissues to further explore their effects on intestinal health. The results showed that the contents of acetate, propionate, and total SCFAs were significantly increased in the BP and AM groups compared to the CON group (*p* < 0.05), while the content of butyrate was also significantly increased in the AM group (*p* < 0.05) (Figure 6A). The expression of the GPR43 gene was significantly higher in the AM and BP groups than in the CON group (*p* < 0.05). The expression of the GPR109A gene was significantly higher in the AM group than in the CON and BP groups (*p* < 0.05) (Figure 6B).

To assess the effect of SCFAs on inflammatory signaling pathways, RT-qPCR and WB were used to determine the mRNA and protein expression content in colon tissues. As shown in Figure 6C,D, the AM group significantly reduced the relative expression of genes and proteins of TLR4 and MyD88 compared with the CON group (*p* < 0.05). In addition, P-NF-κB p65 in the AM group was significantly lower than in the CON and BP groups (*p* < 0.05). At the same time, we determined the relevant expression of colonic inflammatory factors. The results showed that the expression levels of *IL-1β* and *TNF-α* genes were significantly decreased in the AM group compared with the CON group (*p* < 0.05), while the expression level of *TNF-α* genes was also decreased in the BP group (*p* < 0.05); the relative expression of *IL-10* genes was significantly increased in the AM groups (*p* < 0.05) (Figure 6E).

Finally, the relationship between SCFAs and colonic inflammatory factors and their associated pathways, serum immunity, oxidation, and the intestinal barrier was analyzed by Spearman’s correlation analysis (Figure 6F). Correlation analysis showed that total SCFAs and butyrate were significantly positively correlated with the anti-inflammatory cytokine IL-10 (*p* < 0.05). In contrast, they were significantly negatively correlated with pro-inflammatory cytokines (IL-1β, TNF-α), TLR4, MyD88, and P-NF-κB p65 (*p* < 0.05). In addition, total SCFAs and butyrate showed a significant positive correlation with the oxidative index T-AOC and a significant positive correlation with intestinal-barrier-related genes *Claudin1* and *Occludin* (*p* < 0.05). Thus, the results suggest that SCFAs, especially butyrate, are essential in alleviating intestinal inflammation, enhancing antioxidant capacity, and improving intestinal barrier function through the TLR4/MyD88/NF-κB signaling pathway.

## 4. Discussion

Weaned piglets usually suffer a strong stress response due to immature intestinal development, insufficient passive immunity, and nutritional and environmental changes. In this situation, piglets on corn–soybean meal diets still develop diarrhea, resulting in dysbiosis of the intestinal flora and immune barrier dysfunction [7,9]. However, DF plays an active role in alleviating weaning stress and improving intestinal health by regulating the composition of bacterial communities and metabolite production. Moreover, pigs are highly similar to humans in anatomy, physiology, nutrient metabolism, and microbial ecosystem [16]. Therefore, utilizing weaning stress as a model of inflammation to investigate the ameliorative effects of DF on gut health may be an effective nutritional modification strategy to ameliorate microecological dysregulation and gut dysfunction in infants and young children.

### 4.1. DF Relieves Stress and Improves Growth Performance in Weaned Piglets

DF can be classified into SDF and IDF based on its solubility, where microbes can rapidly ferment SDF but also increase digestive viscosity, thus decreasing nutrient utilization. On the contrary, IDF is fermented slowly but increases the intestinal transit rate [6]. Therefore, different sources of DF may be inconsistent in terms of intestinal digestion absorption and regulation of growth performance. In this study, BP contains a large amount of SDF, accounting for about 26%; on the contrary, the content of IDF in AM is higher, accounting for about 96%. However, the AM group significantly increased ADG and decreased F:G compared with the CON group. The BP group showed no significant changes, suggesting that IDF contributed more to the growth performance of piglets than the SDF. Shang et al. [9] showed that TDF was more conducive to promoting piglet growth than SDF, which was consistent with the results of this study. In addition, Yan et al. [8] found that adding BP had no significant effect on the growth performance (ADG, F:G, and body weight) of weaned piglets. Similarly, studies have reported that adding AM improves ADG and feed conversion in weaned piglets [13,21]. However, the addition of BP significantly reduced ADFI, probably because BP as SDF (mainly pectin) increased the viscosity of the chow and improved intestinal filling and retention time, thereby reducing nutrient absorption and feed intake [22]. Therefore, different sources of DF affect the growth and development of the host, whereas AM is more helpful in promoting the growth of weaned piglets.

Weaning stress leads to severe diarrhea with intestinal inflammation, oxidative damage, and increased intestinal permeability [23]. The present study showed that both the BP and AM groups significantly reduced the diarrhea rate. The addition of BP reduced the diarrhea rate in piglets, which may be due to the hydration properties of BP that are suitable for the fermentation of SCFAs [10]. However, the results of studies on the effect of BP groups on the diarrhea rate are inconsistent, with some studies showing no effect of BP on the diarrhea rate [8,9]. This may be related to the source of BP, the amount added, the experimental conditions, and the physiological state of the pigs [24]. Similarly, the diarrhea rate was significantly reduced in the AM group, which may be attributed to the fact that IDF increases the intestinal transit rate and water-holding capacity of intestinal contents, shortens the fermentation time of non-digested food in the colon, and increases fecal volume [12,13]. Serum cortisol is an essential marker of stress response in piglets, which can be released into the bloodstream via the hypothalamic–pituitary–adrenal axis under physiological stress [18]. The serum cortisol and diarrhea rates of BP and AM groups were significantly reduced, indicating that DF had a certain mitigation effect on weaning stress. The three key indicators of LPS, DAO, and D-LA are widely used as intestinal permeability indicators to evaluate intestinal epithelial barrier function [25]. The addition of AM could significantly reduce the activity of LPS and DAO in serum and alleviate the increase in intestinal permeability and barrier function damage caused by weaning stress. In addition, under stress conditions, piglets will increase demand for energy and nutrients, increasing the F:G [26]. Thus, the addition of AM can alleviate weaning stress and reduce intestinal permeability, which is one of the reasons why AM increases the feed conversion rate.

Cytokines play a crucial role in regulating intestinal inflammation. Lower concentrations of inflammatory cytokines are a significant strategy for maintaining the intestinal barrier and reducing inflammation during stress [26]. Addition of DF has been shown to attenuate levels of inflammatory cytokines in the host [27,28]. IL-1β and TNF-α are the principal pro-inflammatory cytokines engaged in weaning stress, which have been demonstrated to elevate intestinal epithelial permeability, prompt pathological disruption of the intestinal junction barrier, and facilitate inflammatory responses [29]. Previous studies have also found that adding AM to the feed of gestating sows reduced the serum levels of pro-inflammatory cytokines in piglets while increasing the levels of the IL-10 [30]. IL-10 is an anti-inflammatory cytokine that regulates immune and inflammatory responses [31]. In addition, AM increased serum immunoglobulin (IgA, IgG, and IgM) levels, whereas the addition of BP showed no significant change. Similar results were obtained with different DF sources added to weaned piglet diets, with the results of wheat bran containing mainly IDF being consistent with those of AM, and again, there was no change in serum immunoglobulin content in the BP group [9]. Immunoglobulin, as an important component of humoral immunity, enhances the phagocytosis of mononuclear macrophages and inhibits the propagation of pathogenic viruses and microbes, which has an important impact on immunomodulation [32]. One possible reason for the beneficial effect of AM on the immune response is that it contains a variety of active substances, such as alfalfa saponins, alfalfa flavonoids, and alkaloids [11]. Therefore, AM may enhance the immune system by reducing the content of cellular inflammatory factors and increasing the production of immunoglobulins, thereby alleviating the intestinal inflammatory response caused by weaning stress.

The antioxidant capacity of the organism is closely related to the health status of the animal. Under normal conditions, the oxidative and antioxidant systems synergistically regulate the production and elimination of free radicals to maintain their usual levels [33]. When weaning stress occurs, the oxidative balance is disrupted, leading to oxidative stress and decreased animal growth performance [23]. In the present study, the addition of BP and AM significantly increased the levels of T-AOC and SOD, similarly indicating that the addition of DF increased the antioxidant capacity of the organisms [34]. When the level of MDA and ROS increases, it will trigger the increase in oxidative stress levels and tissue cell damage in the whole body. In addition, ROS also stimulates the activation of the NF-κB signaling pathway, produces pro-inflammatory cytokines, and causes an inflammatory response [35]. ROS levels were significantly reduced in the AM group. Adding AM to sow diets produced similar effects and reduced ROS levels in piglet serum [30]. In summary, our results show that different sources of DF play an important role in alleviating oxidative damage caused by weaning stress.

Intact intestinal morphology is fundamental for nutrient digestion and absorption [36]. Immunological or inflammatory damage caused by weaning stress disrupts this intestinal morphology, leading to extensive atrophy of the villi and a reduction in germinal crypts [18]. The results of the effects of adding different sources of DF on gut morphology were inconsistent. It has been shown that the addition of SDF increases the viscosity of the contents, which increases the rate of chorionic cell loss and leads to villus atrophy [6]. In the present study, BP had similar results, as evidenced by villous atrophy and deepening of the crypts. In contrast, jejunal villus height and villus height/villus depth were higher in AM piglets. Similarly, many studies have demonstrated that the addition of IDF to the diet improves small intestine morphology [9,37]. Shang et al. [22] found that jejunal villus height and crypt depth were significantly greater in wheat bran added at 6% to the basal diet than in the sugar beet meal. The increase in villus height/villus depth implies an increase in nutrient absorption efficiency [33], which further validates the higher growth performance and feed conversion ratio of AM.

As an essential defense mechanism for regulating and maintaining environmental homeostasis in mammals, the intestinal epithelial barrier prevents intestinal damage by pathogens and their by-products [4]. The epithelial tissue connected by intestinal tight junction proteins is an essential component of the physical barrier of the intestine, preventing the entry of antigens, pathogenic bacteria, and other toxins into the mucosal tissues of the intestinal lumen [38]. Consistent with the present study, it was previously reported that the addition of BP had no significant effect on the expression of intestinal barrier-related genes [9]. In the present study, the addition of AM significantly increased the expression levels of genes related to barrier proteins (including *Claudin-1*, *Occludin*, and *ZO-1*) in the intestine. Previous studies have also shown that adding AM alleviated LPS-induced impairment of intestinal barrier function by increasing the gene expression of Claudin-1 and Occludin [13]. Host defense peptides (HDPs) are small molecule peptides with broad-spectrum antimicrobial effects expressed and secreted by animal tissues and cells, and they are also essential defenses against an invasion of exogenous pathogenic bacteria [39]. *NKlysin*, one of the HDPs, has a wide range of antimicrobial activities, and the addition of DF significantly increased the expression of the *NKlysin* gene. In summary, the results of this study suggest that DF can alleviate diarrhea caused by weaning stress. In addition, AM improves serum immunity, antioxidant capacity, and intestinal barrier function, thereby improving host growth performance.

### 4.2. DF Alleviates Gut Microbiota Dysbiosis in Weaned Piglets and Increases SCFAs-Producing Bacteria

The gut microbiota is involved in host gastrointestinal digestion, immune regulation, and intestinal epithelial barrier integrity maintenance [40]. However, adding DF has been shown to selectively regulate the diversity and composition of the intestinal microbial community, thereby controlling the host’s immune response and metabolism, and benefitting the host [9,12]. Similarly, this study found that adding DF significantly improved the structure of microbes. In particular, the microbial structure separation between AM and other groups was apparent, and the distance was the farthest, indicating that the added microbes of AM had undergone significant structural changes. Subsequently, we performed detailed analyses of ileal and colonic microbes.

In the ileum, there were significant differences in the composition and function of the microbiota between the CON and AM groups. The addition of AM enriched many beneficial microbes, such as *Leuconostocaceae*, *Weissella* and *Pediococcus*, which have antioxidant, anti-inflammatory, and immunomodulatory properties [13,41,42]. In addition, *Actinobacillus* was significantly enriched in the CON group, which promotes the production of LPS, which induces intestinal inflammation and impairs the intestinal barrier function [43]. Moreover, phylogenetic evolutionary trees showed that *Actinobacillus* was more closely related to *Escherichia-Shigella*, a common pathogenic bacterial pathogen [44], further confirming that piglets in the CON group were severely affected by weaning stress. Further, correlation analysis also demonstrated the positive effects of increased beneficial microbes on intestinal health. Therefore, AM can improve intestinal health and promote the digestion and absorption of nutrients by improving piglets’ homeostasis in the ileal microbial environment.

In the colon, the DF group is rich in many fiber-degrading bacteria. At the family level, the AM group’s *Oscillospiraceae* and *Christensenellaceae* were significantly elevated, which play a crucial role in maintaining the structure and function of the gastrointestinal tract by degrading DF to produce SCFAs [45,46]. At the genus level, fiber-degrading bacteria such as *Christensenellaceae_R-7_group* [47], *Pediococcus* [13], and *Weissella* [42] in the AM group were significantly enriched. *Christensenellaceae_R-7_group* also inhibits inflammatory responses by modulating the MyD88 pathway [48]. In addition, *Pediococcus* is significantly enriched in the ileum and plays essential roles in alleviating intestinal inflammation and improving intestinal health. *Weissella* is a probiotic that promotes DF fermentation and produces a variety of beneficial substances, such as extracellular polysaccharides with antioxidant, anti-inflammatory, and immunomodulatory properties [42]. *Pediococcus* can ferment DF to produce SCFAs, which inhibits NF-κB activity and reduces TNF-α production, thereby attenuating LPS-induced inflammatory responses [13]. *Eubacterium_xylanophilum_group* was significantly enriched in the BP group as SCFAs-producing bacteria [49], promoting the production of the anti-inflammatory cytokine IL-10 [50], consistent with the results of this study. In addition, adding DF reduced the relative abundance of the potentially pathogenic bacteria *Colidextribacter* and *norank_f__Selenomonadaceae*. *Colidextribacter* was significantly positively correlated with pro-inflammatory factors, negatively correlated with antioxidant enzyme activities and anti-inflammatory factors [51], and significantly reduced the abundance of *Colidextribacter* in the colon in weaned piglet diets supplemented with probiotics Clostridium butyricum [18]. In addition, there are few reports on *norank_f__Selenomonadaceae*, which belongs to the family level *Selenomonadaceae* and is enriched in patients with episodes of cholangitis [52]. Correlation analysis also showed that *norank_f__Selenomonadaceae* and *Colidextribacter* were significantly negatively correlated with serum immune indexes (IgG, IgM), antioxidant index T-AOC, and intestinal-barrier-related indexes (ZO-1, Occludin). In addition, *Eisenbergiella* enriched in the CON group was also highly correlated with intestinal inflammation and has been validated in several models of inflammatory bowel disease [53]. Thus, DF improves gut health by increasing the number of beneficial microbes, especially fiber-degrading and SCFAs-producing bacteria, and decreasing the number of harmful microbes.

In summary, it was shown that the regulatory effects of different DFs on intestinal microbiota are inconsistent. However, adding DF increased the content of beneficial microbes, especially the significant enrichment of SCFAs-producing bacteria. Correlation analysis showed a significant positive correlation with intestinal health indicators, revealing that DF has a positive effect on alleviating intestinal inflammation, antioxidant, and barrier damage induced by weaning stress, further predicting that this ability of DF may be closely related to the production of SCFAs.

### 4.3. DF Improves Gut Barrier Function by Alleviating Gut Inflammatory Pathways via Microbial-Derived SCFAs

As one of the primary fermentation products of DF, SCFAs are involved in maintaining intestinal cell function and are an important source of energy for colon cells [54]. They are also essential for regulating intestinal cell metabolism, microbial flora, and gene expression. They enhance the intestinal barrier function by influencing intestinal pH to inhibit the growth of harmful bacteria and modulate the immune response. However, the specific mechanisms through which SCFAs protect the intestinal barrier function are complex.

The addition of DF significantly increased the levels of SCFAs in the gut, which, similar to previous results, was associated with the enrichment of fiber-degrading bacteria [13]. In intestinal inflammation, SCFAs, as potential bioactive molecules, can be essential in initiating immune response and regulating mechanisms by binding to receptors on corresponding cells to repair intestinal mucosa and reduce inflammatory damage [14]. However, different SCFAs can bind and activate different types of receptors and thus functions, with acetate, propionate, and butyrate able to start GPR41/GPR43, and GPR109A only activated by butyrate [55]. In this study, *GPR43* and *GPR109A* gene expression was significantly increased by the addition of DF. Acetate and propionate have been shown to alleviate intestinal inflammation by activating the GPR43 receptor and inhibiting the expression of inflammatory factors (IL-1β and TNF-α) [56]. GPR109A, as a receptor for butyrate, acts as a blocker of LPS-induced NF-κB activation, thereby mediating the protective effect of intestinal cells against inflammatory bowel disease [57]. Therefore, DF produces SCFAs through microbial fermentation, which further activates GPR43 and GPR109A receptors on the surface of intestinal epithelial cells, thereby alleviating intestinal inflammation and improving intestinal barrier function.

In addition to GPR receptors, pathogen-initiated immune responses can also be initiated by pattern recognition proteins of the innate immune system (e.g., TLR2 and TLR4), which are significant players in the inflammatory response [58]. TLR4, a typical pattern recognition receptor in the TLR family of proteins, protects the immune homeostasis of the mucosa. NF-κB is a critical molecule in co-regulating inflammatory response and oxidative stress. An essential adaptor molecule for TLR and NF-κB signaling is MyD88 [59]. LPS can be recognized explicitly by TLR4; then, activated NF-κB translocates to the nucleus and induces the expression of various proinflammatory cytokines [60]. However, the activation of TLR4 is conservative, and the SCFAs in the host intestinal tract conform to the signal receptor spectrum of conservative TLR4, inhibiting the large-scale inflammatory response of local TLRs [61]. Therefore, we assessed the expression levels of key factors of this pathway to investigate whether SCFAs could alleviate intestinal inflammation by inhibiting the TLR4/MyD88/NF-κB pathway. The levels of TLR4/MyD88/NF-κB-related genes and proteins in the AM group were significantly decreased, indicating that the pathway was activated, and the expression levels of downstream factors TNF-α and IL-1β mRNA were also decreased, confirming that AM alleviates intestinal inflammation through this pathway. However, excessive production of cytokines has been shown to impair the tight junction function of intestinal epithelial [62]. Subsequently, correlation analysis found that butyrate, in particular, correlated more with intestinal health indicators, indicating that it played a more critical role in inhibiting inflammatory pathways to improve the intestinal barrier. It has also been reported that butyrate can alleviate the inflammatory response by inhibiting the activity of NF-κB [63]. However, the BP group had no significant effect on this signaling pathway, possibly related to the lack of significant changes in butyrate content. In conclusion, AM can improve weaning stress-induced intestinal inflammation and protect the intestinal barrier by improving gut-microbiota-derived butyrate to inhibit the TLR4-MyD88-NF-κB signaling pathway, thereby maintaining intestinal health and improving growth performance.

## 5. Conclusions

**Figure 7:**
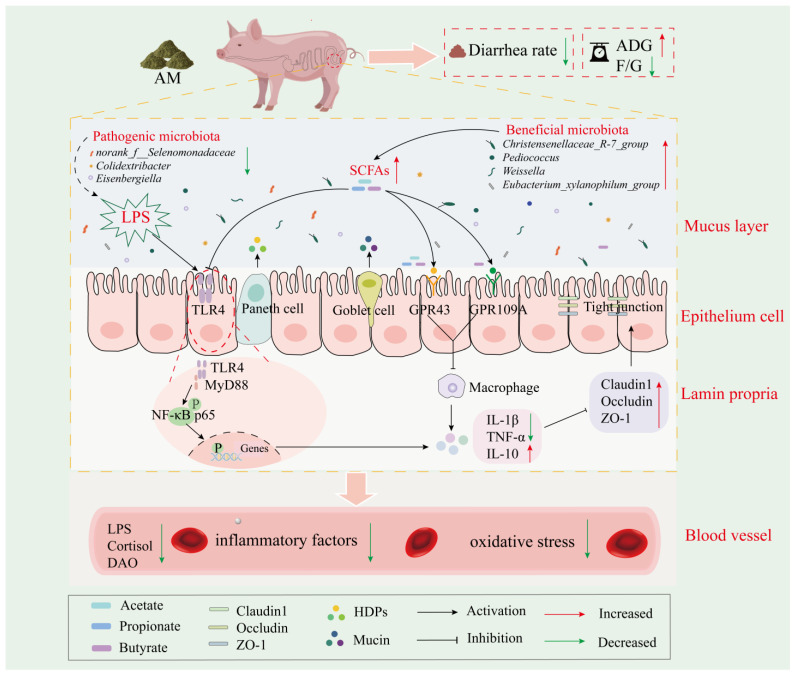
AM alleviated weaning stress by inhibiting the activation of the TLR4/MyD88/NF-κB pathway.

In this study, we investigated the critical microbial-level-mediated roles of different sources of DF in intestinal inflammation and intestinal barrier modulation and their mechanisms, using weaned piglet stress as a classical inflammatory model. The results showed that different sources of DF reduced the diarrhea rate and ameliorated intestinal inflammation and oxidative damage in weaned piglets. Importantly, AM fiber inhibits the activation of the TLR4-MyD88-NF-κB signaling pathway by modulating gut-microbiota-derived SCFAs, thereby modulating intestinal barrier function and improving growth performance. In summary (Figure 7), our results can provide more valuable theoretical support for applying AM to alleviate weaning stress and improve early gut dysfunction, which may have implications for human infants.

## Figures and Tables

**Table 1:** Ingredients and nutrient levels of the experimental diets (g/kg, as fed basis, %).

Item	CON	BP	AM
Ingredients			
Corn	58.40	53.23	53.27
Soybean-puffed	12.00	11.53	11.85
Fermented soybean meal	5.40	5.58	3.99
Soybean meal	10.00	10.00	10.00
Beet pulp meal	-	5.00	-
Alfalfa meal	-	-	5.00
Fish meal	4.00	4.12	4.36
Dried whey	5.00	5.00	5.00
Soybean oil	1.50	1.99	3.15
Salt	0.35	0.32	0.33
Limestone	0.68	0.63	0.60
Calcium hydrogen phosphate	1.00	0.95	0.79
L-Lysine	0.32	0.30	0.32
DL-Methionine	0.14	0.15	0.14
Zinc oxide	0.20	0.20	0.20
* Premix	1.00	1.00	1.00
Total	100.00	100.00	100.00
Nutrient levels			
Digestion energy (MJ/kg)	14.61	14.61	14.61
Crude protein	19.05	19.05	19.05
Crude fiber	2.83	3.76	3.76
Lysine	1.38	1.38	1.38
Methionine + Cystine	0.76	0.76	0.76
Calcium	0.76	0.76	0.76
Available phosphorous	0.39	0.39	0.39

* The premix provided the following per kg of diets: Vitamin A, 5500 IU; Vitamin D_3_, 500 IU; Vitamin E, 66.1 IU; Vitamin B_12_, 28.2 μg; Riboflavin, 5.1 mg; Pantothenic acid, 12.6 mg; Nicotinic acid, 29.8 mg; Choline, 540 mg; Mn, 40 mg; Zn, 120 mg; Fe, 130 mg; Cu, 150 mg; Co, 1 mg; Se, 0.25 mg; I, 4.5 mg.

**Table 2:** Effects of different sources of DF on growth performance of weaned piglets.

Item	CON	BP	AM	*p* Value
Initial body weight, kg	8.74 ± 0.09	8.75 ± 0.26	8.75 ± 0.18	1.00
Final body weight, kg	25.15 ± 0.35 ^ab^	24.32 ± 0.27 ^b^	26.31 ± 0.59 ^a^	0.03
ADFI, kg/d	0.85 ± 0.01 ^a^	0.79 ± 0.01 ^b^	0.83 ± 0.01 ^a^	0.01
ADG, kg/d	0.47 ± 0.01 ^b^	0.44 ± 0.01 ^b^	0.50 ± 0.01 ^a^	0.01
F:G	1.81 ± 0.04 ^a^	1.77 ± 0.01 ^a^	1.66 ± 0.02 ^b^	0.02

ADFI = average daily feed intake; ADG = average daily gain; F:G = feed-to-gain ratio. Results are shown as mean ± SEM. Different superscript letters within a row means significantly different when *p* < 0.05. n = 4.

## Data Availability

The original contributions presented in the study are included in the article/Supplementary Materials, further inquiries can be directed to the corresponding author.

## Supplementary Materials

The following supporting information can be downloaded at: https://www.mdpi.com/article/10.3390/nu16111714/s1, Table S1: Chemical composition of BP and AM (g/kg, as fed basis); Table S2: Specific primers of related genes; Figure S1: Effects of different sources of DF on host defense peptides; Figure S2: Effects of different sources of DF on the ileal dilution curve; Figure S3: Effects of fiber sources on ileal microbiota; Figure S4: Effects of different sources of DF on the colonic dilution curve; Figure S5: Effects of fiber sources on colon microbiota.
