# Supplementary material for: Dietary Fiber-Derived Butyrate Alleviates Piglet Weaning Stress by Modulating the TLR4/MyD88/NF-κB Pathway
Source: Nutrients. 2024 May 30;16(11):1714. doi: 10.3390/nu16111714 (PMC11174469; doi:10.3390/nu16111714)
Supplement: Supplementary file 1 [file nutrients-16-01714-s001.zip › nutrients-2993711-supplementary.pdf]

Table S1. Chemical composition of BP and AM (g/kg, as fed basis).

Item	BP	AM
Dry matter	908.2	923.5
Crude protein	100.8	185.8
Neutral detergent fiber	404.5	380
Acid detergent fiber	205.8	276
Total dietary fiber	611.2	656
Soluble dietary fiber	160.9	23.7
Insoluble dietary fiber	450.3	632.3

Table S2. Specific primers of related genes

Gene	Accession number	Primer sequence (5′→ 3′)
IL-1β	NM_214055.1	Forward: TGATGAAAGATAACACGCCC<br>Reverse: CTGCTTGAGAGGTGCTGAT
TNF-α	NM_001065.4	Forward: CACCACGCTCTTCTGCCTACT<br>Reverse: CGGCTTTGACATTGGCTACA
IL-10	NM_214041.1	Forward: CTTGTCAGAGATGATCCAGTTTT<br>Reverse: TTCACCTCCTCCACGGC
ZO-1	XM_021098896.1	Forward: CAACACACGGTGACGCT<br>Reverse: CAACATTATCCATTGAAACTCC
Occludin	NM_001163647.2	Forward: GCACCCAGCAACGACAT<br>Reverse: ACGCCTCCAAGTTACCACTG
Claudin1	NM_001013611.2	Forward: CTACTCGTCCAACGGGAAAG<br>Reverse: GCAGGAGCAGCAAAGTAG
Muc1	XM_021089730.1	Forward: CGGGCTTCTGGGACTCTT<br>Reverse: GTGCCTGCTTCCGCTTT
Muc2	XM_021082584.1	Forward: CTGTGTGGGGCCTGACAA<br>Reverse: AGTGCTTGCAGTCGAACTCA
Nklysin	NM_001278755.1	Forward: GCCTCATCTGTGAGTCTTGTCG<br>Reverse: CAGTGTCCTCGTTGGGTTGTG
pBD2	NM_214442.2	Forward: TGACTGTCTGCCTCCTCTCTTCC<br>Reverse: GGTCCCCCCTTTCTTGGCAC
REGIIIγ	NM_001144847.1	Forward: GCTTCCTACTGCTATGCCTTG<br>Reverse: ACTGAGCACAGATGCGAGG
GPR41	NM_001315601.1	Forward: GTCTGTGCCCTCATGGGTTT<br>Reverse: GACGTTCATACCTTCGGCCT
GPR43	XM_005664467.3	Forward: CCTGACGCTGGCAGACCT

		Reverse: GCTGCTGTAGAAGCCGAAACC
GPR109A	XM_021072989.1	Forward: AGCCATCATCTCCTGCCTCCTG<br>Reverse: ATCATGCCAGCGGAAGGTATTGC
TLR4	NM_001030693.1	Forward: TGCCCCTACTCAATCTCTCT<br>Reverse: CAAGTTTTCATTACATCCGAAC
MYD88	NM_001099923.1	Forward: TGGAACAGACCAACTATCGGC<br>Reverse: CATCAGAGACAACCACTACCATCC
GAPDH	NM_001206359.1	Forward: CGTCCCTGAGACACGATGGT<br>Reverse: GCCTTGACTGTGCCGTGGAAT

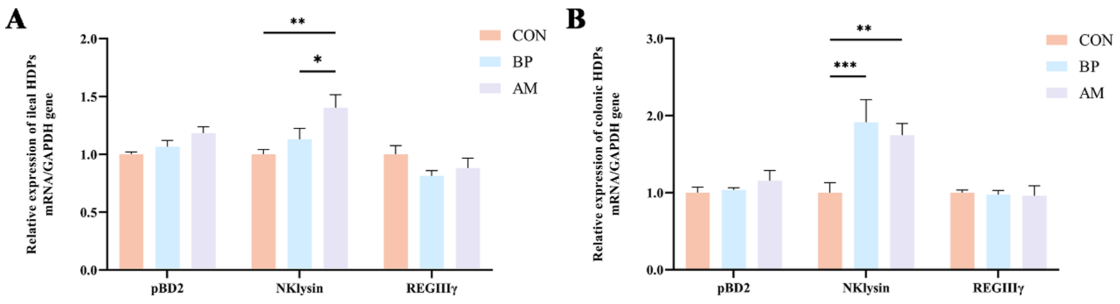

Figure S1. Effects of different sources of DF on host defense peptides (A) The relative gene expression levels of host defense peptides in ileal tissues. (B) The relative gene expression levels of host defense peptides in colon tissues. Data are expressed as the Mean ± SEM. *P < 0.05; **P < 0.01; ***P < 0.001. n=4.

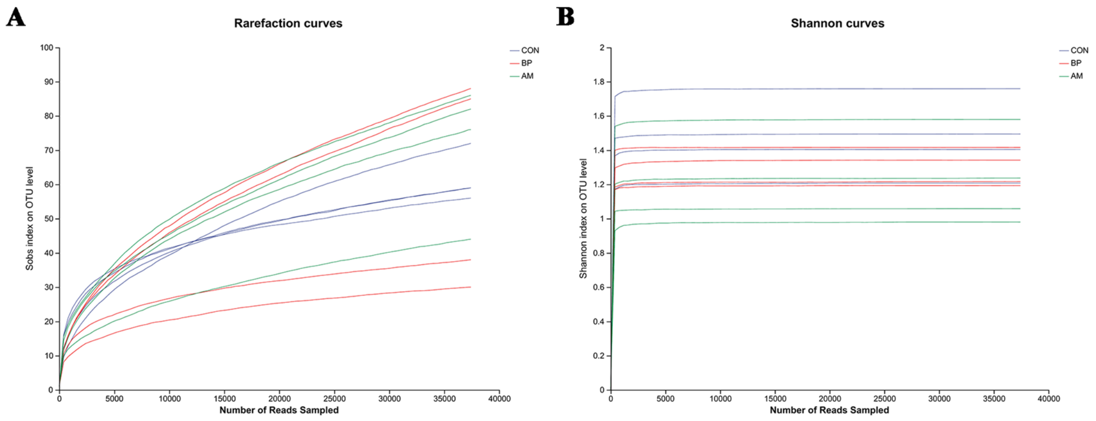

Figure S2 Effects of different sources of DF on the ileal dilution curve. (A) Sobs index on OTU level. (B) Shannon index on OTU level. n=4.

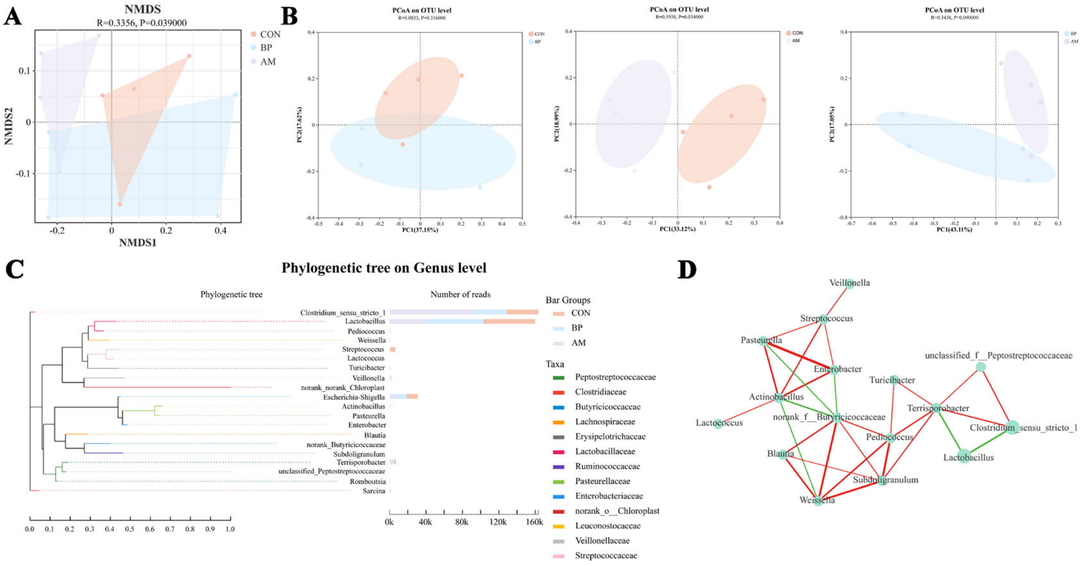

Figure S3. Effects of fiber sources on ileal microbiota. (A) NMDS analysis of microbial composition. (B) PCoA analyzes the composition of the gut microbiota between the three groups. (C) Phylogenetic evolutionary tree showing relationships between the top 20 microbiota. (D) The heat map of the correlation network between the top 20 microbiota and the green line indicates a negative correlation. n=4.

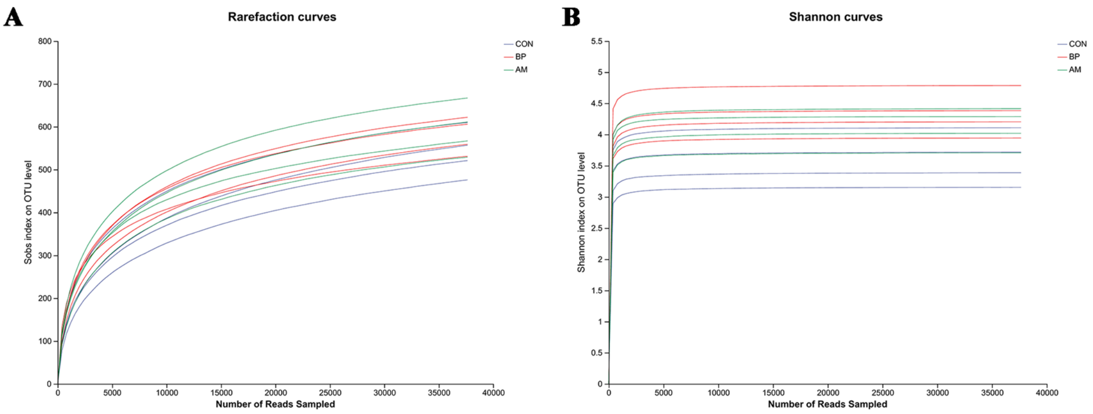

Figure S4 Effects of different sources of DF on the colonic dilution curve. (A) Sobs index on OTU level. (B) Shannon index on OTU level. n=4.

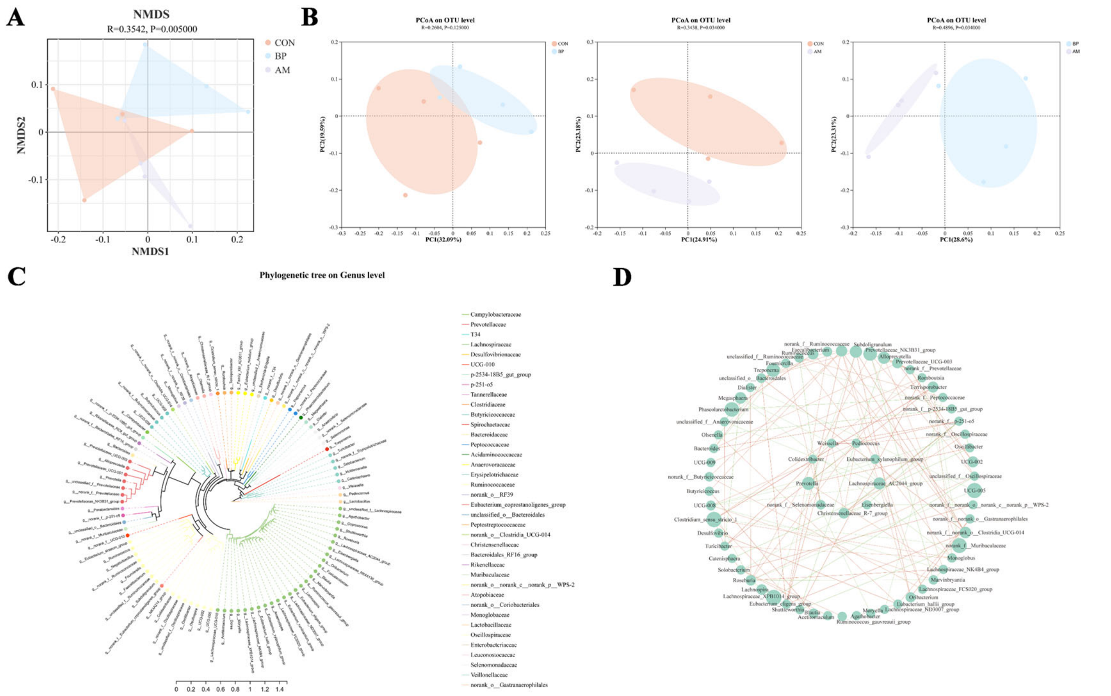

Figure S5. Effects of fiber sources on colon microbiota. (A) NMDS analysis of microbial composition. (B) PCoA analyzes the composition of the gut microbiota between the three groups. (C) Phylogenetic evolutionary tree showing relationships between the top 100 microbiota. (D) The heat map of the correlation network between the top 100 microbiota and the green line indicates a negative correlation. n=4.
